# Isotopic niche provides an insight into the ecology of a symbiont during its geographic expansion

**DOI:** 10.1093/cz/zoab013

**Published:** 2021-02-24

**Authors:** Enrique González-Ortegón, Marta Perez-Miguel, Jose I Navas, Pilar Drake, Jose A Cuesta

**Affiliations:** Instituto de Ciencias Marinas de Andalucía (ICMAN-CSIC), Campus Universitario Rio San Pedro, Avda. República Saharaui, 2, 11519, Cádiz, Puerto Real, Spain; Unidad Asociada Crecimiento Azul CSIC-IFAPA, El Puerto de Santa María Spain, Spain; Instituto de Ciencias Marinas de Andalucía (ICMAN-CSIC), Campus Universitario Rio San Pedro, Avda. República Saharaui, 2, 11519, Cádiz, Puerto Real, Spain; Unidad Asociada Crecimiento Azul CSIC-IFAPA, El Puerto de Santa María Spain, Spain; Unidad Asociada Crecimiento Azul CSIC-IFAPA, El Puerto de Santa María Spain, Spain; Instituto de Investigación y Formación Agraria y Pesquera, IFAPA – Centro Agua del Pino, Ctra. El Rompido-Punta Umbría, km 3.8, 21459 El Rompido, Huelva, Spain; Instituto de Ciencias Marinas de Andalucía (ICMAN-CSIC), Campus Universitario Rio San Pedro, Avda. República Saharaui, 2, 11519, Cádiz, Puerto Real, Spain; Unidad Asociada Crecimiento Azul CSIC-IFAPA, El Puerto de Santa María Spain, Spain; Instituto de Ciencias Marinas de Andalucía (ICMAN-CSIC), Campus Universitario Rio San Pedro, Avda. República Saharaui, 2, 11519, Cádiz, Puerto Real, Spain; Unidad Asociada Crecimiento Azul CSIC-IFAPA, El Puerto de Santa María Spain, Spain

**Keywords:** bivalves, commensal, kleptoparasitism, parasitism, stable isotope, trophic position

## Abstract

The study of the recent colonization of a symbiont and its interaction with host communities in new locations is an opportunity to understand how they interact. The use of isotopic ratios in trophic ecology can provide measurements of a species’ isotopic niche, as well as knowledge about how the isotopic niches between symbiont and host species overlap. Stable isotope measurements were used to assess the sources of carbon assimilated by the host species (the bivalves *Mytilus galloprovincialis* and *Scrobicularia plana*) and their associated symbiont pea crab *Afropinnotheres monodi*, which occurs within these bivalves’ mantle cavities. The mixing model estimates suggest that all of them assimilate carbon from similar sources, particularly from pseudofaeces and particulate organic matter in this symbiotic system based on filter feeding. The symbiotic species occupy comparable trophic levels and its association seems to be commensal or parasitic depending on the duration of such association. The pea crab *A. monodi* reflects a sex-specific diet, where males are more generalist than the soft females because the latter’s habitat is restricted to the host bivalve. The high isotopic overlap between soft females and *M. galloprovincialis* may reflect a good commensal relationship with the host.

The trophic dependence of symbionts on their hosts plays an important role in determining the association pattern of symbiotic systems (Thiel and Baeza 2001). At present, a wide variety of marine crustaceans have been described as symbionts of other macroinvertebrates around the world (Hay et al. 2004). A symbiotic system involves a close and long-term biological interaction between different biological organisms: a host and one or several symbionts which interact in a variety of ways such as sharing or competing for the same resources (Caulier et al. 2014). The effects of symbionts on the mortality and metabolism of their hosts should be analyzed to determine the type of their interactions (Britayev and Lyskin 2002). However, that approach is too difficult for many symbiotic relationships because it requires a lot of time and special equipment. A closely related matter of study, which is also relevant to assess the nature of a given association may be the type of trophic relationship established between a host and its symbiont (Martin and Britayev 1998).

Trophic ecology can provide knowledge about how these species interact, for instance, morphological and functional analysis of the food procuring system and the digestive tract, direct observation of the feeding, and analysis of the intestinal contents (Martin and Britayev 1998). However, what we know about feeding preferences and trophic relationships of symbionts is still relatively limited (Boecklen et al. 2011; Gonçalves et al. 2020). Previous studies have showed that symbionts: might assimilate similar food (e.g., Kennedy et al. 2001); or the same food as the host or steal from the food resource of the host species, kleptoparasitism (Martin et al. 2002); could occupy a higher trophic level than their host due to their capacity for food selection (Cabanellas-Reboredo et al. 2010); assimilate diverse external food sources (Caulier et al. 2014) such as organic matter in suspension (Vandenspiegel et al. 1992); feed on host mucus with no effects on the host species (Poore and Bruce 2012); or feed on host tissues (Britayev and Lyskin 2002; Parmentier and Das 2004). The study of these trophic relationships is vital to an understanding and an assessment of the impact of symbionts on host communities, especially when a symbiont exhibits a higher geographical expansion rate to new latitudes, thus invading their native communities.

The spread of nonindigenous species to new ecosystems is becoming increasingly common in many regions of our planet (Vitousek et al. 1997). In the current scenario of rising seawater temperatures, marine symbionts exhibit a higher geographical expansion rate to new latitudes (Drake et al. 2014): for instance, the African pea crab *Afropinnotheres monodi* Manning, 1993, as well as other African marine invertebrates, are experiencing a geographical expansion of range in European waters driven by climate change (e.g., González-Ortegón et al. 2020). Among marine symbionts, the brachyuran crabs of the family Pinnotheridae De Haan, 1833 display a wide diversity of host–guest interactions (Drake et al. 2014; Castro 2015; Perez-Miguel et al. 2019a); among these, the species of the subfamily Pinnotherinae De Haan, 1833 are characterized by living as ecto or endosymbionts with invertebrates, mainly bivalves (Castro 2015; Palacios Theil et al. 2016). In the last 10 years, the pea crab *A. monodi* has exhibited a considerable increase in, and successful settlement of, southern regions of Europe (Perez-Miguel et al. 2018); and has been found inhabiting the mantle cavity of living bivalves of >10 species in Iberian waters, with a high prevalence in *Cerastoderma edule* (Linnaeus, 1758) and *Mytilus galloprovincialis* Lamarck, 1819, and with a lower prevalence in *Scrobicularia plana* (da Costa, 1778), and *Ruditapes decussatus* (Linnaeus, 1758) (Drake et al. 2014; Perez-Miguel 2018; Perez-Miguel et al. 2019a). Like other pea crabs (Becker and Türkay 2010), *A. monodi* usually has a facultative free-living stage in both sexes (males and hard females), whereas reproductive females (soft females) have a last obligate and sedentary parasitic stage in their host (i.e., organisms permanently living on hosts), where males usually visit them.

The study of the trophic relationships of this symbiont and its interaction with the native bivalve host communities is necessary to understand the impact of this new arrival to the Gulf of Cadiz. The abundance of *A. monodi* varies among the populations of host species and the type of symbiotic relationship between the pea crab and the bivalves is not completely clear, although some deleterious effects (reduced gills), as well as a loss of condition in *M. galloprovincialis*, have been found (Drake et al. 2014; Perez-Miguel et al. 2018). Despite the apparent importance of the pea crab—bivalve symbiosis, the effects of the invasive pea crab on the native host bivalves are unknown.

Many successful free-living non-native species are classified as trophic generalists (Marvier et al. 2004), characterized by their wide ecological tolerance and diets, allowing them to be highly successful in new habitats (Sax and Brown 2000). However, obligate symbionts differ from free-living organisms in that they depend on strong biotic interactions with their hosts, which alter their niche and spatial dynamics (Mestre et al. 2020). In the case of endosymbionts permanently living on hosts, it is hard to prove the feeding habits and the type of biological interaction. The stable isotopes of nitrogen (δ^15^N) and carbon (δ^13^C) could demonstrate whether a symbiont is sharing sources and occupying a similar trophic level and provide a powerful tool to estimate the trophic positions (TPs) of, and carbon flow to, non-native consumers in food webs (see McCue et al. 2020). Variation in naturally occurring stable isotopes can be used to study food web dynamics in diverse ecosystems and has long been appreciated in general ecology (Martinez del Rio et al. 2009). The stable isotopes have the added advantage that they provide longer-term estimates than acute behavioral observations or stomach content analyses; are mainly well suited to detect subtle differences in resource use patterns in marine habitats where it is difficult to observe animals directly (Cherel and Hobson 2007; Soto et al. 2019). This is also true for crustaceans where the digestion of prey in the stomach can make it difficult to ascertain trophic relations among species in an ecosystem (González-Ortegón et al. 2015). In addition, the use of isotopic ratios in trophic ecology can provide measurements of the isotopic niches where symbiont and host species overlap (Guzzo et al. 2013). A species’ isotopic niche refers to the multivariate space in which the axes are the isotopic values for different elements occupied by the isotopic composition of an animal’s tissues (Newsome et al. 2007; Martinez del Río et al. 2009). A recent review about stable isotope analysis shows that parasites have largely been neglected compared to other trophic levels such as carnivores (Boecklen et al. 2011).

In general, few studies have analyzed trophic relationships using stable isotope analysis between symbiont invertebrates and host bivalves by comparing the spatial variation in diet breadth and overlap of symbiont species (Kennedy et al. 2001; Cabanellas-Reboredo et al. 2010; Caulier et al. 2014). In pinnotherids, there are only 2 previous studies, the early work by Kruczynski (1975) using radioisotopid techniques for the study of the feeding of *Tumidotheres maculatus* (Say, 1818) (as *Pinnotheres maculatus*), and the more recent study by Cabanellas-Reboredo et al. (2010), which extends the work by Kennedy et al. (2001) applying mixing models to the C and N stable isotopes to study the trophic association of *Nepinnotheres pinnotheres* (Linnaeus, 1758) with its host, *Pinna nobilis* Linnaeus, 1758.

Symbionts often inhabit the same spaces, share the same host, and interact in a variety of ways such as sharing or competing for the same resources. According to the niche theory, 2 or more species cannot permanently and simultaneously occupy exactly the same foraging niche if resources are limited (Hutchinson 1957). However, when the isotopic overlap between the endosymbiont and the host organisms is high, both species could be living with a high abundance of food, or the symbiont could be consuming residual food left by the host (in that case, the host would not be affected). Experimental studies manipulating the quantity of food or even forcing the symbiotic system to food stress could provide relevant information to assess the interaction and the effect of the African pea crab on its host bivalves. For instance, under conditions of limited food, although the symbiotic systems could show a different behavior, the information obtained would be relevant to test whether the host is affected. Further, if the species feeds on its host, fewer individuals may be able to cohabit on a single host than in a species that only utilizes its host for protection (Thiel and Baeza 2001).

The study of the type of food resources exploited by the African pea crab *A. monodi*, how isotopic niches of a symbiont and its hosts overlap and how this overlap varies among host species are relevant aspects to know the impact of this African species on the native host communities and the configuration of the symbiont’s niche. In addition, it is important to investigate how the sex influences the isotopic niche of this symbiont, since soft females are permanent endosymbionts and males and hard females show a facultative free-living stage. In this study, we explore the trophic interactions of the African pea crab by combining an experimental approach with field work in the Gulf of Cadiz: 1) experiments were conducted in the laboratory to determine whether the pea crabs would predate mussels under conditions controlling food and 2) stable isotope data from field samples were analyzed to investigate the carbon sources, TPs, the breadth of the trophic niche, and the degree of diet overlap between the symbiont pea crab *A. monodi* and 2 of its host bivalves from the Gulf of Cadiz. Finally, we used stable carbon isotope ratios to investigate whether food use differed between uninfected and infected bivalves. Infection by the pea crab caused a loss of condition in the host species *M. galloprovincialis* (Perez-Miguel et al. 2018). By altering the condition of the host population, as well as resource use, these non-lethal effects of parasitism could alter energy transfer in littoral food webs. Knowledge of these interactions would provide important insights into how symbionts and host species coexist and could contribute to management strategies, including conserving commercially valuable native bivalves potentially threatened by interactions with the African pea crab.

Niche widths can vary for different host species, as well as depending on the sex of the symbiont, which could show sex-related differences in foraging niches considering the particularities of the life cycle of the pea crab (Perez-Miguel 2018). The alternative hypotheses about the symbiont’s expected diet, and its potential implications for the type of interaction would be: (1) the African pea crab feeds on the host’s pseudofaeces (particles wrapped in mucus by the bivalves and expelled without having passed through its digestive tract) and, in consequence, the role of the symbiont is to clean the host bivalve from the excess food, (2) the African pea crab consumes similar food to the host and thus both species compete for resources, and (3) the symbiont predates on host muscle, displaying a parasitic behavior. Although we could hypothesize that this invasive crab species is feeding on the living bivalve (parasitism), it would not be consistent if the association is only commensalism or kleptoparasitism as already defined for other pea crab species (Orton 1920; Iyengar 2008). At the same time, we hypothesized that, since the availability of prey should be similar and the ranges of the study species overlap, the symbiont species’ patterns of isotopic niche area, and trophic interactions would remain constant across different host species.

This study applies contemporary stable isotope approaches to assess the trophic ecology and niche overlap of the pea crab *A. monodi* and host species. Naturally, occurring stable isotopes of carbon (δ^13^C) and nitrogen (δ^15^N) reveal distinct aspects of a consumer’s long-term trophic niche by providing a time- and space-integrated representation of dietary carbon sources and relative TP (Layman and Allgeier 2012). Recently, quantitative metrics and statistical frameworks have been developed to examine stable isotope variation among defined groups to understand trophic diversity and quantify niche space (Jackson et al. 2011; Layman et al. 2012; Ausems et al 2020). These tools allow for a better characterization of the ecological role of generalist species and their potential effects on native species and their food webs (Guzzo et al. 2013).

## Materials and Methods

### Study area and sampling

**Figure 1.**
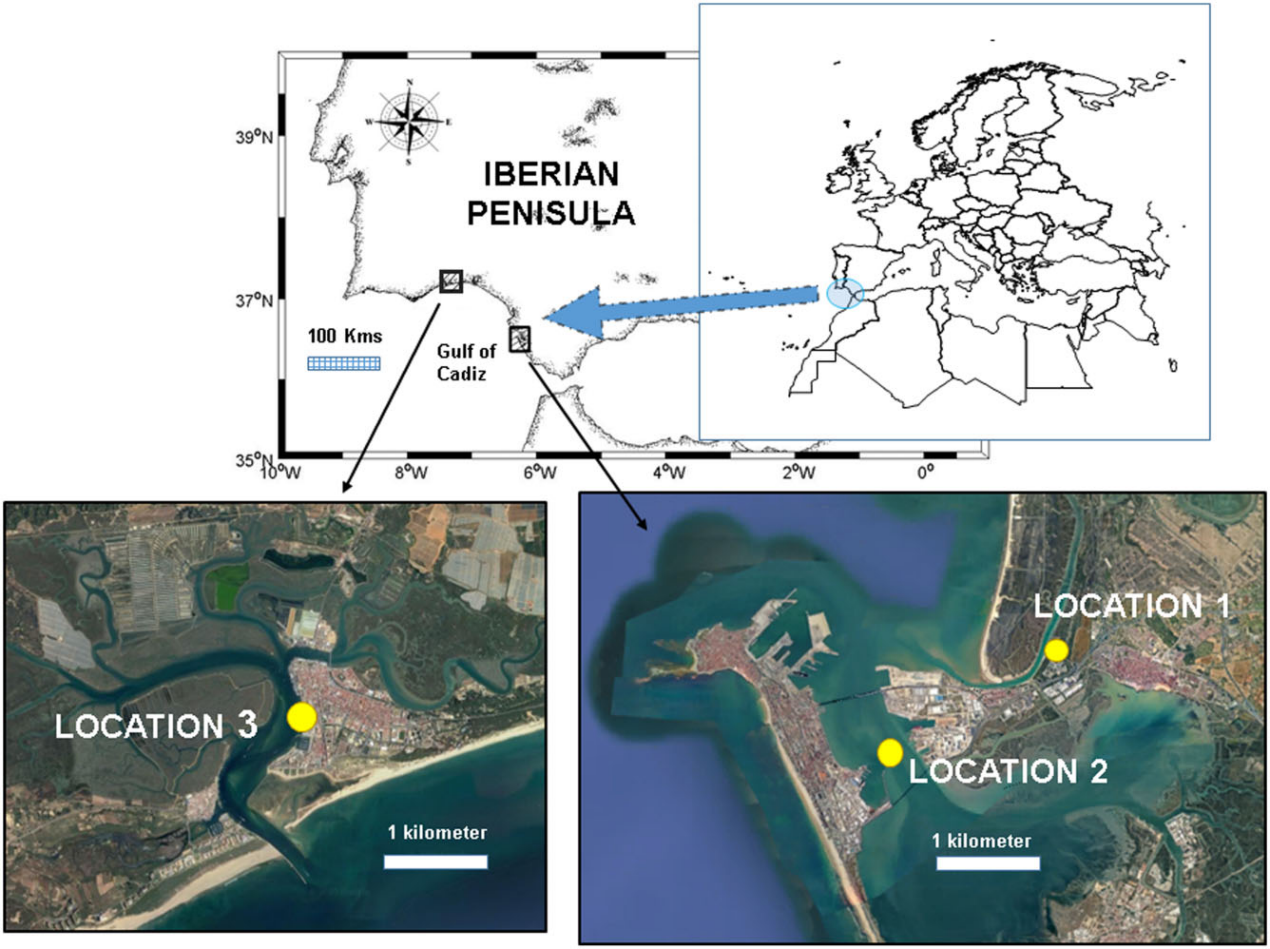
Map of the Iberian Peninsula and the 3 locations sampled of the Gulf of Cadiz: (1) San Pedro estuary (Cádiz) for the collection of clams (*S. plana*); (2) Cádiz Bay, and (3) Carreras estuary Huelva for the collection of mussels (*M. galloprovincialis*).

This study was conducted in the south-west of Europe (Gulf of Cadiz: 37°24′18″N, 6°51′48.5″W), where the African pea crab was collected in 1995 (López de la Rosa et al. 2002) for the first time in European waters. The effect of the African pea crab *A. monodi* on bivalve species has been studied since 2010 (Drake et al. 2014). Two host bivalve species, the clam *S. plana* and the mussel *M. galloprovincialis* with a low and high infestation rate, respectively, were collected in 3 locations in the Gulf of Cadiz in the spring of 2018 (Figure 1). A stable population of the clam *S. plana* inhabits the intertidal mud flats of the Río San Pedro inlet, where clams burrow relatively deep into the sediment of the high tide zone. The mussel *M. galloprovincialis* can be found all around the Gulf of Cadiz, attached to artificial hard structures (see Drake et al. 2014 for details). *Scrobicularia plana* individuals were sampled at Location 1, situated in the intertidal zone of the mouth of the San Pedro estuary; and those of *M. galloprovincialis* in Locations 2 and 3, in the subtidal zone within the Bay of Cadiz and in the intertidal zone of the Carreras estuary (Huelva), respectively (Figure 1). Considering the variability of the infestation rates in previous studies (Drake et al. 2014; Perez-Miguel et al. 2019b), ∼100 individuals of each bivalve species were collected to assure the presence of the symbiont crab in the samples. Intertidal bivalves at Locations 1 and 3 and the subtidal mussels in Location 2 were also collected by hand by scuba divers from the submerged chains of the most infested buoy (Buoy 12) found within the Bay of Cadiz (see Cuesta et al. 2020 for details).

### Monitoring experiment

For the monitoring experiment under controlled feeding conditions, the host species chosen was the mussel *M. galloprovincialis* because it was the bivalve species mainly used as a host by the only obligatory symbiont phase of *A. monodi* (soft female). A total of 290 mussel individuals from Location 3 (Carreras estuary and Huelva) were controlled in the laboratory to limit the types of prey available for both the mussel and the crab *A. monodi* (living inside) and to test whether the pea crabs would consume any mussel tissue over time. The mussels were collected in spring of 2018 from a breakwater of the Carreras river estuary and deposited in experimental tanks (40 L) in 2 different batches. Water flow rates in the tanks were high (160 mL min^−1^), resulting in an exchange rate of 620% day^ − 1^. The average temperature was 19.6°C. Microalgae were added to the tanks at a uniform rate and, the concentration of microalgae was quite similar to what is found in natural conditions (120,000 cel mL^−1^). The tanks were cleaned every week to prevent bacterial developments. Feeding experiments were conducted for a total of 30 days in the tanks. Forty animals were sampled (20 mussels per tank) on Days 0, 7, 14, and 30 and, after this period, they were under starvation and sampled again on Days 37 and 51. Thus, a total of 240 mussels were dissected with a mean size of 47.6 mm (range size of 39.3–58.2 mm). The infestation rate of pea crabs in each sample varied between 48% and 52%.

During the starvation period, the animals were not fed to force a potential predation of the pea crab on the host bivalve.

### Food sources and sample preparation for stable isotope analysis

Samples of the organic matter were collected to estimate the proportion of the sources contributing to the consumers’ diet in the mixed models. Particulate organic matter (POM) and microalgae from marine origins were sampled by collecting water from the open sea. For each water sample, a volume of 5 L of sub-surface water was sampled by passing it through a 200 μm mesh for POM, and then vacuum filtering through pre-combusted GFF filters. Macroalgae were collected because a significant fraction of macroalgal biomass is integrated in sediments in coastal ecosystems, and thus the contributions of green algae, such as *Ulva* sp. Linnaeus, 1753, to the diets of suspension-feeding species could not be excluded (Dubois et al. 2007c). *Ulva* sp. was collected close to the bivalve sampling sites by hand. At Location 3, 2 co-occurring suspension-feeding species, *Magallana gigas* (Thunberg, 1793) and *Balanus* sp. Costa, 1778, were collected to validate the TPs of the host bivalve species and partially integrate the isotopic food source in the field. Although feeding mechanisms could partly explain the inter-specific differences in trophic niches, similar differences in TPs would be expected in individuals colonizing the same space and receiving the exact same food mixture from the water column (Dubois and Colombo 2014). Finally, pseudofaeces were collected, when they were available from the host bivalve species as a feasible food source (Kennedy et al. 2001).

Upon collection, all the sources were frozen, the shell length of each host bivalve was measured to the nearest 0.1 mm with a dial calliper (Tesa Cal IP65), and opened for tissue dissection; the symbiotic crab was removed when it was present. Muscle in the *S. plana and M. galloprovincialis* individuals from the field (Locations 1 and 2, respectively) were separated and used for stable isotope analysis. In the case of the mussels, *M. galloprovincialis* collected at Location 3, different tissues (muscle, mantle, gill, gland, and gonad) were separated as potential sources both from the field and during the experiment and analyzed separately. Tissues from the host bivalves, the pea crabs’ muscle tissue, *Ulva* sp., and muscle tissue of *M. gigas* and *Balanus sp*. were rinsed in distilled water before being oven dried at 50°C for 24 h. POM samples on the GFF filters were treated with concentrated HCl to remove any carbonates, and subsequently re-dried. All samples were homogenized, weighed in tin cups and analyzed for carbon and nitrogen content and stable isotope ratios using an elemental analyzer (Carlo Erba CHNSO 1108) coupled to an isotope-ratio mass spectrometer (Finnigan MAT Delta Plus) at the Stable Isotope Facility, University of Coruña. The analytical precision [standard deviation (SD), *n* = 10] was 0.15‰ for both N and C, as estimated from standards analyzed along with the samples. These laboratory standards were previously calibrated according to international standards supplied by the International Atomic Energy Agency (IAEA, Vienna).

The stable isotope ratios in the samples are expressed as delta notation (δ, ‰), deviations from the isotopic ratios found in Vienna Pee Dee belemnite (δ^13^C) and atmospheric nitrogen (δ^15^N). When the C: N ratios were >3.5, the muscle tissue samples were corrected for lipid content as this was found to influence the δ^13^C values (Post et al. 2007). For pseudofaeces where the C or N content was so low that it decreased the precision of the isotopic analysis, the mean values pooled across samples from the same location and their SD were used instead. Among the discrimination values available in the literature, we chose to use 2.2‰ for δ^13^C and 3.8‰ for δ^15^N, since these values were specifically calculated using feeding experiments for suspension feeders such as *M. edulis* Linnaeus, 1758 (Dubois et al. 2007a, 2007c; Lefebvre and Dubois 2016). As variability in trophic discrimination, we included the associated error estimates in fractionation calculations using the propagation of errors in the Dubois et al. (2007a, 2007c) model (that is 0.259 for Δ^13^C and 0.243 for Δ^15^N). We minimized the variability in trophic enrichments, as the host bivalves were similar in size and were maintained under similar and constant environmental conditions in the experiments.

### Statistical analysis of stable isotope data

A multivariate approach to the analysis of location in the isotopic composition of the host bivalves and pea crabs was followed using the Plymouth Routines in Multivariate Ecological Research version 6.1 computer software pack. Multivariate data analysis was carried out by Euclidian distance similarity for isotopic data calculated on the fourth root-transformed data (Clarke and Gorley 2006). ANOSIM (Analysis of similarities) tests were carried out to determine significant differences among locations in the dual, δ^13^C and δ^15^N isotopic signatures in the host bivalves and pea crabs. We tested for the effects of uninfected and infected bivalves (Supplementary Table S1, Model 1), the feeding experiment (bivalves reared under ad libitum food condition vs. starvation period) in mussels (Supplementary Table S2, Model 2) and in the soft female (Supplementary Table S2, Model 3) pea crabs by fitting different statistical models. In Models 1 and 2, we considered the effects of bivalve size and the different mussel tissues (muscle-mantle-gill-gland-gonad) using an ANCOVA (Analysis of Covariance) type model with a PERMANOVA test (version 6, Primer-E Ltd., Plymouth, UK).

The MixSiar Bayesian stable isotope-mixing model (Semmens et al. 2009; Stock and Semmens 2016) was used to determine probability distributions for the proportional contribution of the food sources (POM, microalgae, detritus, and bivalves) to the diet of each pea crab. Location was included in the analyses as a random effect. This analysis was performed in the “R” environment (R Development Core Team 2018). We used 3 Markov Chain Monte Carlo (MCMC) chains to fit the mixing model. The prior information used was uninformative, which represents the same probability for all sources (Stock and Semmens 2016) and the error structure used was “Residual * Process” (Parnell et al. 2010; Stock and Semmens 2016). The MixSIAR mixing model parameterization included 3 chains, a chain length of 1,000,000, burn‐in interval of 500,000, and thin‐by interval of 500. Results are reported as medians with 95% Bayesian credible intervals (see Table 1). The Bayesian procedures within the MixSIAR framework include statistical diagnostic tests to assess MCMC convergence. The Gelman–Rubin test is based on analyzing multiple simulated MCMC chains by comparing the variance within each chain to the variance between chains (Gelman et al. 2003). Large deviation between these variances indicates non‐convergence and the ratio will be near 1 at convergence. The Geweke test is a 2‐sided z‐test comparing the mean of the first part of the chain with the mean of the second part. At convergence, these means should be the same, and large absolute z‐scores indicate that the result should be rejected.

**Table 1.** Mean ± SD of δ^13^C and δ^15^N values in the pea crab *A. monodi* and its prey from 3 different locations in the area studied: (1) San Pedro estuary (Cádiz); (2) Cadiz Bay; (3) Carreras estuary (Huelva)

Host	Location	δ^13^C** **±** **SD (‰)	δ^15^N** **±** **SD (‰)	*n*
*S. plana*	1	**−19.70 ± 0.25**	**11.14 ± 0.43**	31
*A. monodi*		**−19.66 ± 0.75**	**10.28 ± 0.73**	22
Macroalgae		**−**17.39 ± 2.08	6.60 ± 0.82	13
Pseudofaeces		**−**19.47 ± 0.01	6.29 ± 0.12	2
Microalgae		**−**24.43 ± 0.07	0.04 ± 0.01	30
POM		**−**22.43 ± 0.22	6.04 ± 0.91	10
*M. galloprovincialis*	2	**−19.77 ± 0.41**	**9.58 ± 0.49**	7
*A. monodi*		**−18.75 ± 1.68**	**9.38 ± 0.33**	8
Macroalgae		**−**17.39 ± 2.08	6.6 ± 0.82	13
Pseudofaeces		**−**21.48 ± 0.02	8.52 ± 0.14	5
Microalgae		**−**24.43 ± 0.07	0.04 ± 0.01	30
POM		**−**21.38 ± 0.85	5.81 ± 1.34	10
*M. galloprovincialis*	3	**−18.18 ± 0.23**	**7.67 ± 0.42**	17
*A. monodi*		**−19.33 ± 1.25**	**6.61 ± 0.86**	58
Macroalgae		**−**17.39 ± 2.08	6.6 ± 0.82	13
Pseudofaeces		**−**21.48 ± 0.02	8.52 ± 0.14	5
Microalgae		**−**24.43 ± 0.07	0.04 ± 0.01	30
POM		**−**21.38 ± 0.85	5.81 ± 1.34	10

Values of the pea crab and host in bold. Prey values are not corrected for isotopic discrimination. *n*, number of individuals analyzed for δ^13^C and δ^15^N values.

The TPs of the pea crabs and the host bivalves were calculated using Bayesian estimations from consumer stable isotope ratios and the tRophicPosition package (Quezada‐Romegialli et al. 2018) in the “R” environment (R Development Core Team 2018). Two trophic baselines were included in the model: organic matter from marine suspended POM and microalgae, and from green macroalgae as a proxy of benthic detritus. The MCMC simulations were run through 10,000 iterations and a burn-in of 10,000 with 5 chains.

Niche widths and overlap for δ^13^C and δ^15^N were determined using the Stable Isotope Bayesian Ellipses package (Jackson et al. 2011) in the ‘R’ environment (R Development Core Team 2018). As a measure of foraging niches, we calculated posterior ellipses (siberMVN) for δ^13^C and δ^15^N for both species with 2 × 104 iterations, a 1 × 103 burning, thinned by 10 and >2 chains. We used uninformed priors, as we had no prior knowledge of our expected results. We determined the size of the niche width of each group using Bayesian Standard Ellipse Areas (BSEAs, siberEllipses) and then used Bayesian Overlap to calculate the niche overlap area between the corresponding Bayesian Estimates for the 95% Prediction Ellipse Area (BEPEA). As we were interested in how much of each individual niche overlapped with the others, niche overlap was calculated as the proportion of overlapping BEPE relative to the BEPE of each group separately (Ausems et al. 2020).

## Results

### Monitoring experiment in the symbiotic system

The stable isotope values, the carbon and nitrogen content, and C/N ratio in mussels did not differ significantly between those infected or uninfected with the African pea crabs (Supplementary Tables S1 and S2). The stable isotope values of the mussels showed a significant trend with size.

When we compared the isotopic composition, carbon and nitrogen content, and C/N ratio in the mussels reared under ad libitum food during 30 days (Day 30) and the individuals that continued the culture with nonaccess to food during 21 days (Day 51), we did not find significant differences (Supplementary Table S3, Model 2 and Supplementary Table S4). However, the starvation period appeared to reduce the carbon and nitrogen content in the African pea crabs (Supplementary Table S3, Model 3 and Supplementary TableS4).

### Isotopic composition of the sources and the symbiont as predator

**Figure 2.**
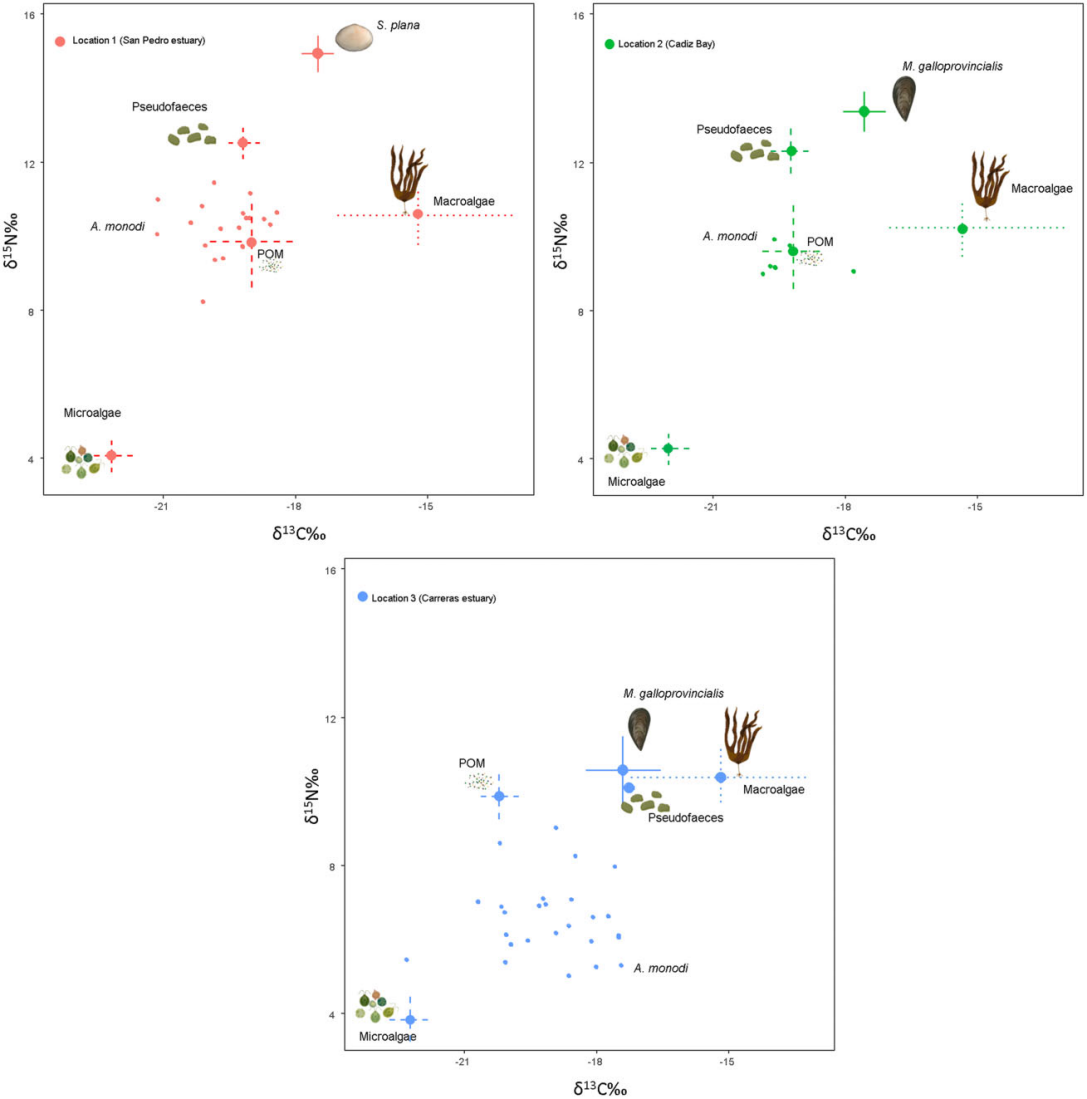
Position of *A. monodi* (small circles inside ellipses) in dual isotope space relative to its potential prey items (mean** **±** **SD** **=** **dashed lines) at 3 locations of the Gulf of Cadiz: (1) San Pedro estuary (Cadiz), (2) Cadiz Bay, and (3) Carreras estuary (Huelva). The trophic discrimination factors for *A. monodi* muscle were calculated using muscle-specific diet tissue discrimination factors from Dubois et al. (2007a). Location was considered as a random factor. Prey types (Pseudofaeces, Microalgae, , Macroalgae and the bivalves *M. galloprovincialis* and *S. plana*) are depicted by line drawings. For sample sizes, see Table 1.

The bivalve species’ δ^15^N values were slightly enriched compared to those of the pea crab, whereas the δ^13^C values varied slightly between them (Table 1). Inter-individual variability of pea crabs was related to the corresponding host bivalve species variability (Figure 2), and the spatial variation in isotopic compositions was more evident and mainly due to the δ^15^N values (see Supplementary text and Figure S1). Macroalgae *Ulva* sp. exhibited a very large range for δ^13^C values, but the microalgae values showed a narrower range of variation. Interestingly, the pea crab and its host bivalves exhibited a similar isotopic composition, indicating a similar diet at each symbiotic complex (Table 1). In any case, the bivalves were included as a potential source and, after adjusting for trophic enrichment, all the stable isotope values of the pea crab were within the isoscape bounds of the sources used for the Bayesian mixing models (Figure 2).

### Diet estimates for the symbiont

**Figure 3.**
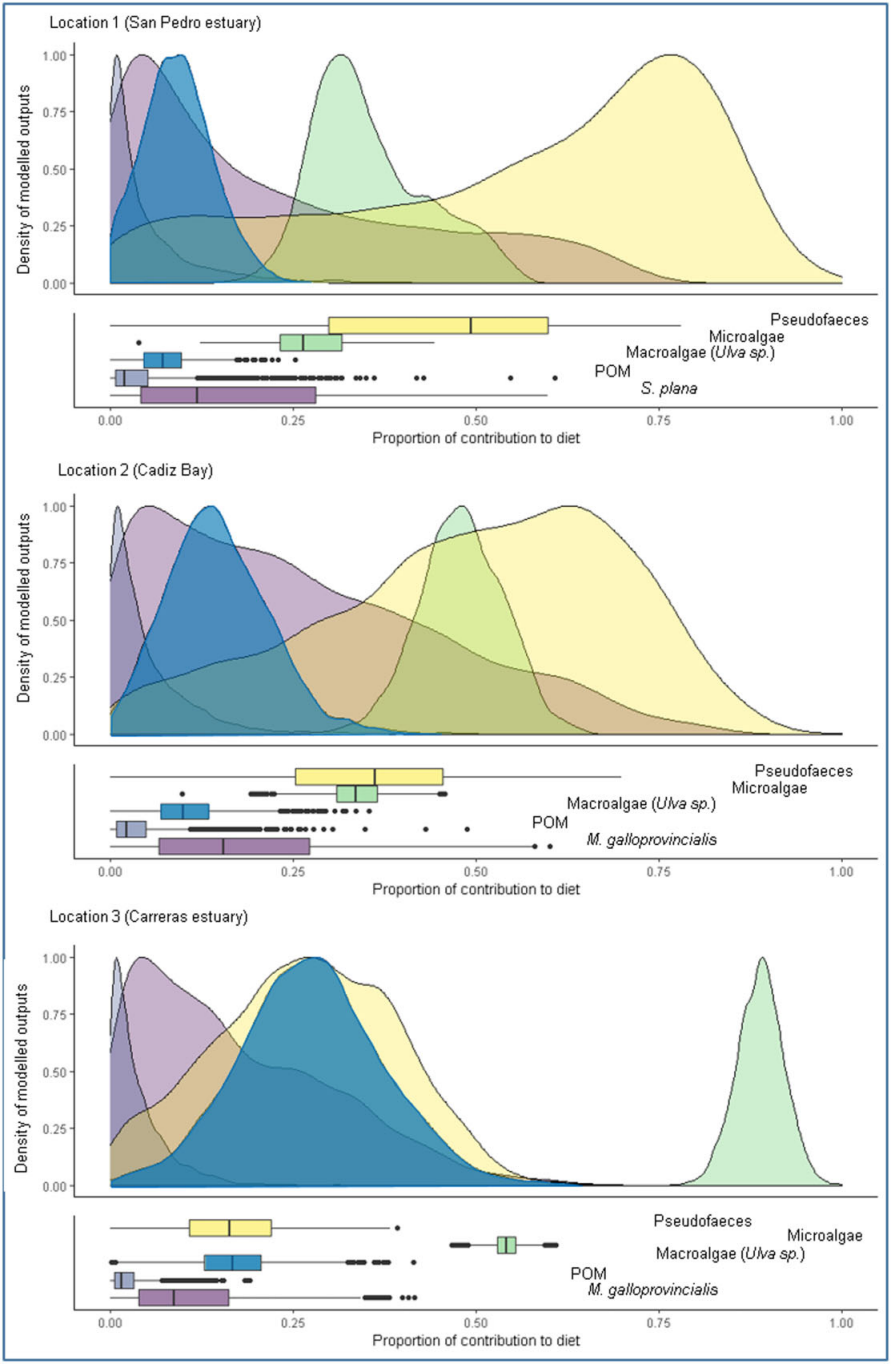
Density distributions and associated boxplots of modeled outputs from Bayesian stable isotope mixing models estimating the proportion of contributions of likely types of diet (Pseudofaeces, Microalgae, POM, Macroalgae, and the bivalves *M. galloprovincialis* and *S. plana*) of the pea crab *A. monodi* at 3 locations of the Gulf of Cadiz (Location 1, San Pedro estuary, Cádiz, Location 2 Cádiz Bay, and Location 3 Carreras estuary, Huelva).

The mixing model estimates indicated that the diet of the pea crab at the 3 locations was dominated by a combination of microalgae and pseudofaeces (>60%; Table 2). The posterior distributions showed an increase in microalgae or a decrease in pseudofaeces from Location 1 in the Bay of Cadiz to Location 3 in Huelva (Figure 3). Overall, the mussels’ muscle tissue showed a low contribution to the pea crab diet (13.3% as potential prey) (Figure 3 and Table 2). It is interesting to highlight that none of the bivalves analyzed showed any damage to their tissues, also during the experiment under the starvation period (individuals from Location 3), with the exception of only 2, out of a total of a 100, that showed reduced gills but without symptoms of picking.

**Table 2.** Bayesian mixing model median estimates (95% CI) of the proportional contributions of each prey type to *A. monodi’*s diet at 3 locations of the Gulf of Cadiz: (1) San Pedro estuary (Cádiz); (2) Cádiz Bay, (3) Carreras estuary (Huelva)

		*S. plana*	*M. galloprovincialis*	
Prey type	Global	San Pedro estuary	Cadiz Bay	Carreras estuary (Huelva)
Bivalves	13.3 (0.6–44.0)	11.9 (0.2–51.8)	15.5 (0.4–48)	8.7 (0.3–28.7)
POM	2.6 (0.1–19.6)	1.9 (0–19.3)	2.1 (0.1–14.9)	1.5 (0–8.8)
Macroalgae	12.3 (3.1–35.1)	7.1 (0.5–15.2)	9.9 (1.8–21.4)	16.7 (5.2–29)
Microalgae	36.4 (13.9–59.2)	26.4 (18.2–41.1)	33.5 (25.4–41.2)	54.2 (50.3–58)
Pseudofaeces	29.9 (5.4–56.7)	49.3 (2.2–69.2)	36.2 (3.8–57.3)	16.3 (1.5–30.1)

### TPs and isotopic niche

**Figure 4.**
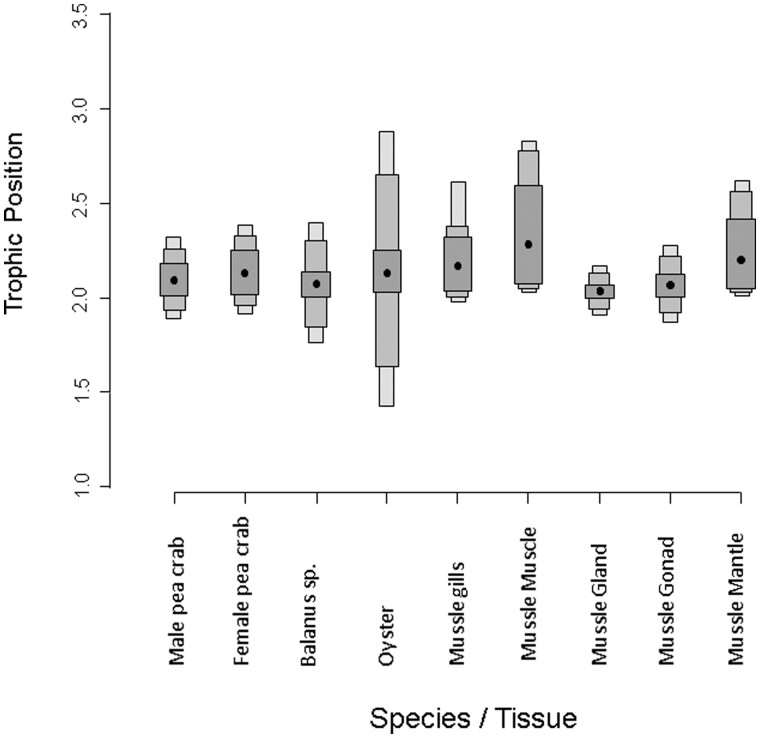
Bayesian posterior TPs for the female and male pea crabs *A. monodi*, the filter-feeding species Balanus sp. and the oyster *M. gigas* (pooled sample) and different tissues of the host mussel *M. galloprovincialis* as calculated from 2 sources Bayesian model at the location of Carreras estuary (Huelva). Overlap of the 95% credible intervals indicates similarity between groups. Gray boxes represent 50% (dark shading), 75% (medium shading), and 95% (light shading) credible intervals, with black dots designating the median.

The (TPs of the mussels and the pea crabs in dual isotope space did not shift substantially, and they were similar to other filter-feeding species such as the Japanese oysters *M. gigas* and *Balanus* sp. (Figure 4).

**Figure 5.**
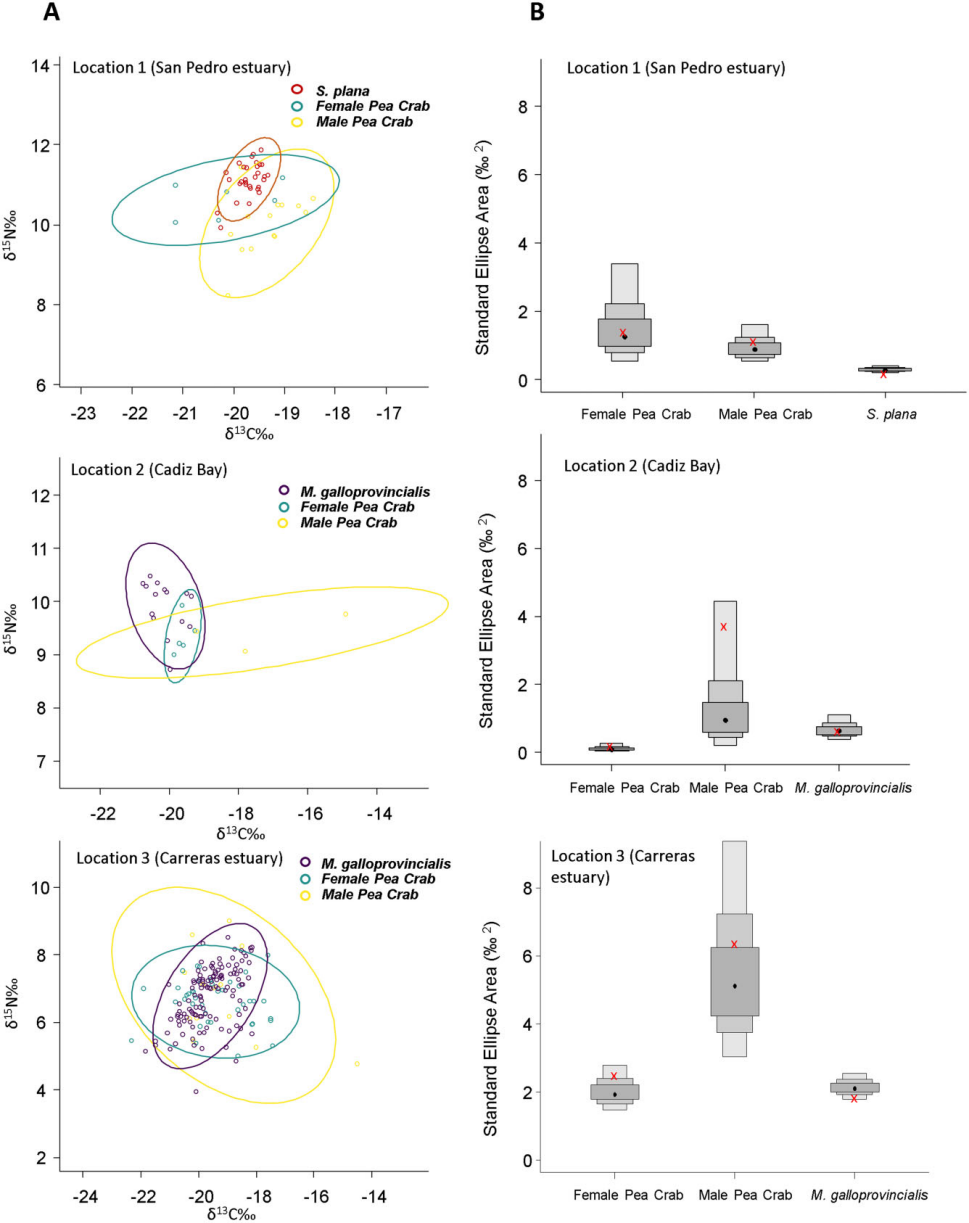
(**A**) Independent calculated ellipses containing 95% of the isotopic data for the male and female of the pea crab *A. monodi* and the host bivalves *S. plana* (Location 1) and *M. galloprovincialis* (Locations 2 and 3) from the Cadiz Bay. (B) Posterior distributions of isotopic niche size (BSEA) for the females and male pea crab *A. monodi* and the host bivalves *M. galloprovincialis* and *S. plana* at 3 locations of the Gulf of Cadiz: (1) San Pedro estuary (Cádiz); (2) Cádiz Bay; and (3) Carreras estuary (Huelva). Gray boxes represent 50% (dark shading), 75% (medium shading), and 95% (light shading) credible intervals, with red crosses designating the model values and black dots the median.

**Figure 6.**
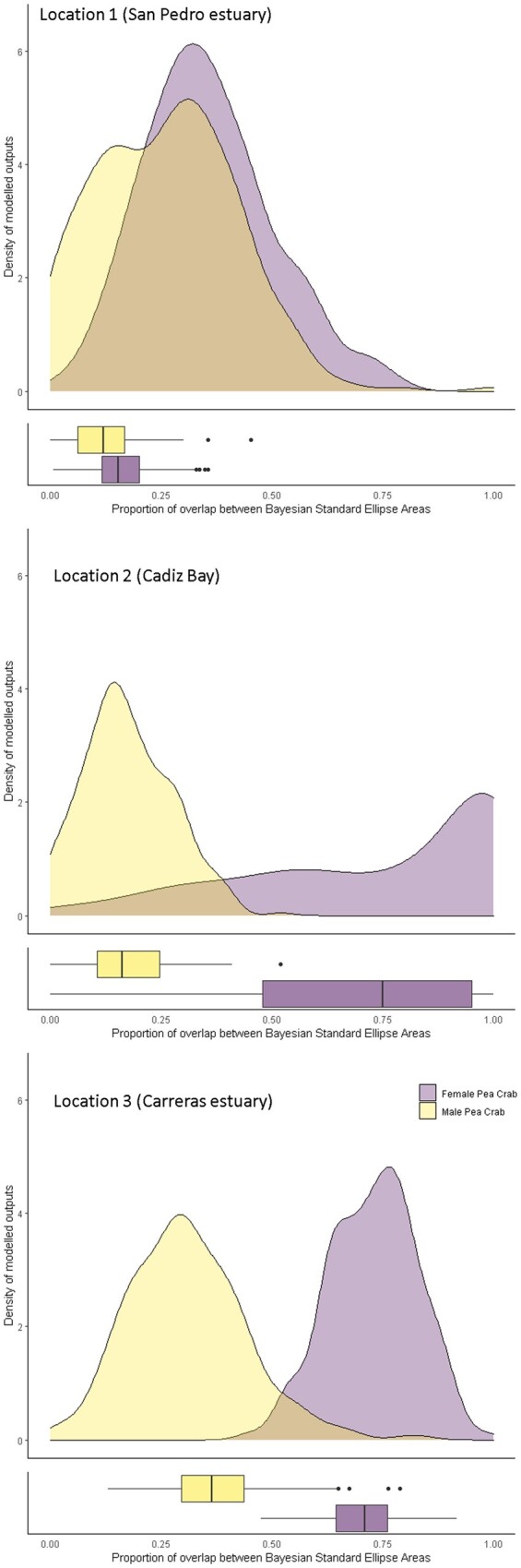
Density distributions of the overlap proportion of BSEAs and associated boxplots between the niche area of the host bivalves and the male and female pea crab *A. monodi* obtained from posterior distributions, at 3 locations of the Gulf of Cadiz: (1) San Pedro estuary (Cádiz); (2) Cádiz Bay; and (3) Carreras estuary (Huelva). Overlap was calculated as the percentage of shared area of each individual ellipse (male or female of the pea crab) with each relevant other ellipse (the host bivalves).

BEPEA overlap analyses revealed that between the 2 host bivalves, the pea crabs showed less overlap in δ13C and δ15N with *S. plana* than with *M. galloprovincialis* (Table 3 and  Figure 5). In the case of *M. galloprovincialis*, the female pea crab had the smallest Bayesian stable isotope standard ellipse area and, therefore, the most specialized trophic niche compared to that of the male pea crab (Figure 5b). Compared to the male pea crabs, the females showed a high overlap with both host bivalves; but it was considerably higher with *M. galloprovincialis* than with *S. plana*, which was negligible (Table 3 and Figure 6).

**Table 3.** Bayesian standard ellipse area overlap for δ^13^C and δ^15^N for the pea crab *A. monodi* (soft females and males) and the host bivalves *M. galloprovincialis* and *S. plana*, at the 3 locations studied

		*S. plana*	*M. galloprovincialis*	
Species versus	Species	San Pedro estuary	Cadiz Bay	Carreras estuary (Huelva)
Female pea crab	Host bivalve	15.39 (4.89–31.48)	83.94 (11.15–100)	70.93 (54.78–84.26)
Male pea crab	Host bivalve	11.95 (0.11–25.32)	16.25 (0–38.39)	36.47 (20.52–59.75)
Host bivalve	Female pea crab	85.88 (47.37–100)	11.98 (1.46–31.05)	66.87 (54.01–83.67)
Host bivalve	Male pea crab	40.17 (0.28–93.05)	32.04 (0–77.95)	99.15 (81.73–100)

Overlap was calculated as the percentage of area shared of each individual’s ellipse with each relevant other’s ellipse. Median and (CI)—credible interval of 95%.

## Discussion

The stable isotopic data reported here demonstrate that the symbiont pea crab *A. monodi* seems not to predate on the host’s muscle (Supplementary Tables S1–S4); the bivalves and the pea crab *A. monodi* assimilate similar food sources, being the diet of the pea crab dominated by a combination of microalgae and pseudofaeces (Figures 2 and 3); the crabs occupy a similar trophic level to its host (Figure 4); with a sex-specific diet, where males are more generalist than the soft females because the latter’s habitat is restricted to the host bivalve (Figure 5); and the biotic environment or the morphology of the host species could determine differences in the isotopic overlap between both species (Figure 6). Therefore, our results support the use of stable isotope data in host–symbiont interaction studies.

The off-host environment, such as the concentration of microalgae, was manipulated in the laboratory and allowed us to observe that the host bivalve was not affected by the symbiont (Supplementary Tables S1–S4). The environmental characteristics of a habitat include biotic and abiotic factors, unrelated to the host, that can directly influence the fitness of a symbiont or indirectly influence a symbiont by affecting the host (Cardon et al. 2011). The bivalve host is probably not significantly affected beyond the removal of its pseudofaeces or, alternatively, theft of its filtered microalgae. Indeed, when the lack of food could be evident (restricted for 20 days in the monitoring experiment), it seems that the carbon and nitrogen content affected the symbiont but not the host (Supplementary Table S2). Carbon and nitrogen are proxies for lipids and proteins, respectively (González-Ortegón et al. 2018). Probably, the host allocated a higher proportion of carbon and possibly a higher proportion of lipid reserves than the symbiont, and under the experimental time of food limitation, the resources available for growth and reproduction were too low for the pea crab soft female (reproductive females). In contrast, previous observations in the field have suggested that, as in other pea crab species (Sun et al. 2006; Trottier et al. 2012; Mena et al. 2014), the soft females of *A. monodi* might compete for food with their host since a reduction in the condition index of mussels hosting soft females has been observed (Perez-Miguel et al. 2018). In coastal waters, the amount of food available may decrease depending on the season (Dubois and Colombo 2014) but would rarely disappear during such a long period as 20 days (as in the monitoring experiment). Probably, under environmental conditions with a low amount of food available the energetic reserves of both the symbiont and the host could be affected, particularly in the host species if the symbiont steals what little amount of food is available to the host species. Frequent situations of low food concentrations in the field, throughout the life of the mussel, could also explain a reduction in the condition index of the host bivalves. While in the forced laboratory conditions without access to food, the species with fewer energetic reserves would be affected first: in this case, the soft pea crab with the main reproductive role.

Previous trophic studies in the host species showed that POM and microphytobenthos might comprise a significant part of the diets of cultivated and wild suspension-feeders such as bivalves (Dubois et al. 2007c). It is commonly assumed that they depend primarily upon suspended particles (i.e., phytoplankton) filtered from the water column and that they ultimately all compete for the same food mixture brought by tides and wave action (Dubois and Colombo, 2014). In the case of the invasive *A. monodi*, the contributions from the different sources modeled for pea crabs were more consistent with a high contribution from microalgae-based particulates and, alternatively, pseudofaeces, when an excess of organic particles accumulated by the host bivalve occurs (Figures 2 and 3). *Afropinnotheres monodi* is not truly filter feeding, since they feed, as other pinnotherids, by brushing the mucus strings from the bivalve gills with their chelipeds (Becker and Türkay 2017). Probably, mucus associated to the labial palps of the bivalves when they generate pseudofaeces could contribute, although poorly, to the diets of the symbiont crab as inferred from Bayesian stable isotope mixing models (Kennedy et al. 2001). Although detritus and macroalgae were not clearly significant in the diet of the pea crab, probably in other seasons, when a significant fraction of macroalgal biomass is incorporated in sediments after being dislodged and fragmented in coastal food webs, they may thus enter the diets of suspension-feeding species (Dubois et al. 2007b). This is in line with other studies that have demonstrated the importance of pseudofaeces and POM in this symbiotic system based on filter feeding (Kennedy et al. 2001; Cabanellas-Reboredo et al. 2010).

The stable isotopic data reported here demonstrate that the bivalves and the pea crab *A. monodi* assimilate similar food sources, with the crab occupying a similar trophic level to its host and other co-occurring suspension-feeding species (Figures 4 and Supplementary Figure S1). A previous study has showed that pea crabs can select food in other host-bivalve species and this may allow them to increase their trophic level (Cabanellas-Reboredo et al. 2010). However, our data indicate that the host-bivalve forages at a slightly higher TP than the pea crab. There is ∼1 ‰ difference between the δ15N of bivalves and the δ15N of pea crabs (Supplementary Figure S1 and Table 1), which indicates it is likely that they occupy similar trophic levels. This observation is consistent with earlier studies, suggesting that the pea crab mainly uses the host for protection (Kennedy et al. 2001). These authors defined that such interspecific association was commensal; but according to the feeding habits of the pea crab, it should basically be referred to a kleptoparasitism since the crabs take, or steal, the food that the host bivalve filters from the water (Becker and Türkay 2017). However, if, due to this feeding behavior, the host suffers damage then this relationship cannot be defined as kleptoparasitism (Mena et al. 2014), and this has been observed in several pinnotherid species (Tablado and Gappa 1995; Mercado-Silva 2005; Trottier et al. 2012).

Overall, the male pea crabs exhibited wider stable isotope niches compared to the females (Figures 5 and 6). The difference in the lifestyles between males, hard females (facultative symbiotic), and soft females (obligate symbiotic) could explain the difference in foraging niche widths. Soft females inhabit the mussel’s mantle cavity permanently (reproductive female) and feed more restrictively on those filter-based components, whereas males are sporadic visitors to this bivalve species only in reproductive periods and feed on a wide variety of food sources. For some free-living crab, detritus and microphytobenthic food sources have been shown to play a primary dietary role (Alderson et al. 2013; Hübneṙ et al. 2015). Thus, the soft female pea crab has the most specialized trophic niche and a higher overlap with the host bivalves and potentially a smaller range of prey types when compared to that of the male pea crab. When we compare isotopic overlap between the pea crab and the host bivalve species, this one was higher in *M. galloprovincialis* than in *S. plana* (Figure 5). Individuals of *S. plana* inhabit intertidal muddy flats and filter only the water closest to the bottom, whereas those of *M. galloprovincialis* filter higher up in the water column. Filter-feeding hosts with generalist diets or superabundant suspended organic particles as preys could allow a high trophic overlap in the symbiotic system (Ausems et al. 2020). This may serve to reduce competition among species, although wild suspension-feeders do not necessarily compete for food, and the feeding mechanisms are of fundamental importance to understand the partitioning of food resources and dietary overlap of co-occurring species (Dubois et al. 2007c). The high isotopic overlap between soft females and *M. galloprovincialis* may reflect a good commensal relationship with the host (Canesi and Pruzzo 2016). The availability of space within hosts is a relevant factor in determining the final size of the sedentary phase (soft females) of pea crabs and, consequently, of their reproductive pattern (Drake et al. 2014; Perez-Miguel et al. 2020). Among host bivalves, the flattened morphology of the shell and the relatively small size of *S. plana* make this host rather unsuitable for the soft females of this symbiont, and this constraint leads to a decrease in size-specific fecundity (Drake et al. 2014). Thus, the biotic environment in the host species determines symbiont survival, reproduction, and population growth (Rohde 1994).

The expansion of marine species into colder waters is expected in the current scenario of rising seawater temperatures. For example, other African crustaceans have expanded their northern limit of distribution in the northern Atlantic waters (e.g., González-Ortegón et al. 2020). Since this symbiotic crab has a solitary life inside bivalves (mainly one crab per host, Drake et al. 2014), it probably has invaded other host bivalves during its geographic expansion to northern latitudes because this diversification of the host species allows it to increase the probability of finding suitable hosts, establishing large populations and decreasing the risk of predation. Like other European pinnotherids *Pinnotheres pisum* (Linnaeus, 1767), *A. monodi* is a generalist in bivalve host use and this feature is favoring its northward expansion (Perez-Miguel et al. 2019b). From an evolutionary perspective, which is not the aim of this study, an adaptation which facilitates biotic interactions with a novel host would expand the fundamental niche of this invasive symbiont species (Guisan et al. 2014; Mestre et al. 2020) and can generate evolutionary novelties and extend the phenotypic niche space (Borges 2017). Further, as the diets of this symbiont in a new host species are similar in composition to the suspension-feeding diet of the host bivalve species (Figure 3 and Table 2), they are largely dependent on the nature and quantity of the organic particles (Dubois and Colombo 2014), and any habitat rich in POM, mainly enriched with microalgae could allow this association. In this sense, the isotopic niche could be an indicator of the match between a symbiont and its host species.

## Supplementary Material

zoab013_Supplementary_Data
