# Supplementary material for: Isotopic niche provides an insight into the ecology of a symbiont during its geographic expansion
Source: Curr Zool. 2021 Feb 24;68(2):185–97. doi: 10.1093/cz/zoab013 (PMC8962723; doi:10.1093/cz/zoab013)

**SUPPLEMENTARY TABLES**

**Isotopic niche provides an insight into the ecology of a symbiont** **during its geographic expansion**

Enrique González-Ortegón^a,b*^, Marta Perez-Miguel^a,b^, Jose I Navas^b,c^, Pilar Drake^a,b^ and Jose A.Cuesta^a,b^

^a^Instituto de Ciencias Marinas de Andalucía (ICMAN-CSIC), Avda. República Saharaui, 2, 11510 Cádiz, Puerto Real, Spain, ^b^Unidad Asociada Crecimiento Azul CSIC-IFAPA, Spain, ^c^Instituto de Investigación y Formación Agraria y Pesquera, IFAPA. Centro Agua del Pino, Ctra. El Rompido-Punta Umbría, km 3.8, 21459 El Rompido, Huelva, Spain

*Address correspondence to Enrique González-Ortegón. E-mail: e.gonzalez.ortegon@csic.es

Handling editor: Zhi-Yun JIA

Received on 20 November 2020; accepted on 13 February 2021

**Supplementary Table S1**.Results of the 3-way PERMANOVA for isotopes (*δ*13C and *δ*15N), C and N content and C/N ratio in the mussel *Mytilus galloprovincialis* for the monitoring experiment. Design with the fixed factors type of tissue (muscle-mantle-gill-gland-gonad), infected or uninfected mussels with the African pea crab *Afropinnotheres monodi* (reared under ad libitum food and starvation period) and the covariate size of mussels (Size) (Model 1). The analysis is based on the modified Euclidean distance dissimilarity (9999 permutations). Significant terms are highlighted in bold.

|  |  |  | Isotopes |  |  |  | C and N content | |  | C/N |  |  |
| --- | --- | --- | --- | --- | --- | --- | --- | --- | --- | --- | --- | --- |
| Model 1 | Source | df | SS | Pseudo-F | P(perm) |  | SS | Pseudo-F | P(perm) | SS | Pseudo-F | P(perm) |
|  | Size | 1 | 9.15 | 14.58 | **2e-4** |  | 5.54 | 0.79 | 0.43 | 0.54 | 2.46 | 0.12 |
|  | Type(Ty) | 4 | 16.13 | 6.42 | **1e-4** |  | 410.58 | 14.66 | **1e-4** | 10.7 | 12.16 | **1e-4** |
|  | Infestation (Inf) | 1 | 0.22 | 0.34 | 0.68 |  | 10.05 | 1.43 | 0.25 | 0.47 | 2.16 | 0.14 |
|  | Ty*Inf | 4 | 1.22 | 0.48 | 0.78 |  | 2.15 | 0.07 | 0.98 | 0.04 | 0.05 | 0.99 |
|  | Res | 38 | 23.85 |  |  |  |  |  |  | 8.41 |  |  |
|  |  |  |  |  |  |  |  |  |  |  |  |  |

**Supplementary Table S2**. Average and standard deviation in δ^13^C, δ^15^N, carbon and nitrogen content and C/N ratio of pooled mussels’ tissues of *Mytilus galloprovincialis* infected and uninfected with the African pea crab *Afropinnotheres monodi*.

|  |  | | | | | | | | | | | | | |
| --- | --- | --- | --- | --- | --- | --- | --- | --- | --- | --- | --- | --- | --- | --- |
| Presence of crabs | δ^13^C | | δ^15^N | | Percentage C | |  | Percentage N | | | C/N | | Size | |
|  | Mean | SD | Mean | SD | Mean | SD |  | Mean | SD | Mean | | SD | Mean | SD |
| No crabs | -18.59 | 0.40 | 6.89 | 0.57 | 35.69 | 2.57 |  | 9.36 | 1.35 | 4.50 | | 0.54 | 51.55 | 2.92 |
| With crabs | -18.53 | 0.59 | 6.72 | 0.91 | 35.12 | 2.61 |  | 8.64 | 1.06 | 4.80 | | 0.66 | 49.87 | 6.23 |

**Supplementary Table S3.** Results of the 3-way (Model 2) and 1-way (Model 3) PERMANOVA for isotopes (*δ*13C and *δ*15N), C and N content and C/N ratio in the mussel *Mytilus galloprovincialis* for the monitoring experiment. Design with the fixed factors type of tissue (Type: muscle-mantle-gill-gland-gonad), Food period (Food: reared under ad libitum food and starvation period) and the covariate size of the mussels (Size). 3-way model: Model 2 (Type-Food-Size) tested in mussels; 1-way model: Model 3 (Food) tested on the African pea crab *Afropinnotheres monodi*. The analysis is based on the modified Euclidean distance dissimilarity (9999 permutations). Significant terms are highlighted in bold.

|  |  |  | Isotopes |  |  |  | C and N content | |  | C/N |  |  |
| --- | --- | --- | --- | --- | --- | --- | --- | --- | --- | --- | --- | --- |
| Model 2 | Source | df | SS | Pseudo-F | P(perm) |  | SS | Pseudo-F | P(perm) | SS | Pseudo-F | P(perm) |
|  | Size | 1 | 9.15 | 16.43 | **1e-4** |  | 1.47 | 0.35 | 0.69 | 0.27 | 0.12 | 0.73 |
|  | Type(Ty) | 4 | 16.13 | 7.23 | **1e-4** |  | 247.75 | 15.02 | **1e-4** | 23.12 | 2.64 | **0.04** |
|  | Food (Fo) | 1 | 1.71 | 3.07 | 0.07 |  | 11.33 | 2.74 | 0.08 | 4.22 | 1.93 | 0.17 |
|  | Ty*Fo | 4 | 2.41 | 1.08 | 0.36 |  | 12.98 | 0.78 | 0.58 | 2.37 | 0.27 | 0.90 |
|  | Res | 38 | 21.16 |  |  |  | 164.85 |  |  | 87.51 |  |  |
| Model 3 | Source |  |  |  |  |  |  |  |  |  |  |  |
|  | Food | 1 | 1.46 | 0.37 | 0.67 |  | 671.78 | 15.23 | **6e-4** | 0.21 | 1.24 | 0.28 |
|  | Res | 17 | 66.25 |  |  |  | 749.66 |  |  | 2.89 |  |  |
|  |  |  |  |  |  |  |  |  |  |  |  |  |

**Supplementary Table S4.** Average and standard deviation in δ^13^C, δ^15^N, carbon and nitrogen content and C/N ratio of pooled mussels’ tissues of *Mytilus galloprovincialis* and the African pea crab *Afropinnotheres monodi* after 30 days reared under ad libitum food (Day 30) and subsequently exposed to no access to food during 21 days (Day 51).

|  | Time | δ^13^C | | δ^15^N | | Percentage C | |  | Percentage N | | | C/N | | Size | |
| --- | --- | --- | --- | --- | --- | --- | --- | --- | --- | --- | --- | --- | --- | --- | --- |
|  |  | Mean | SD | Mean | SD | Mean | SD |  | Mean | SD | Mean | | SD | Mean | SD |
| *M. galloprovincialis* | Day 30 | -18.57 | 0.61 | 6.65 | 0.84 | 35.54 | 2.75 |  | 8.74 | 1.11 | 4.81 | | 0.67 | 50.55 | 5.91 |
|  | Day 51 | -18.48 | 0.45 | 6.95 | 0.87 | 34.64 | 2.21 |  | 8.84 | 1.23 | 4.63 | | 0.60 | 49.45 | 5.63 |
|  |  |  |  |  |  |  |  |  |  |  |  | |  |  |  |
| *A. monodi* | Day 30 | -16.87 | 1.24 | 6.82 | 0.87 | 31.74 | 3.35 |  | 6.01 | 0.67 | 6.16 | | 0.41 |  |  |
|  | Day 51 | -16.50 | 2.06 | 6.41 | 1.17 | 24.88 | 3.37 |  | 4.92 | 1.01 | 5.65 | | 0.41 |  |  |

## Supplementary text: Isotopic variability of the symbiont and the host species

In the present study, differences among the two host bivalve species at the three locations occurred in δ^15^N and these differences were due to the locations of these individuals (Figure 2 and Figure S1). The stable isotope values of the pea crabs among the three locations shifted significantly between locations (R=0.593, p<0.01), and these differences were due to the δ^15^N values (R=0.846, p<0.01), but not due to the δ^13^C values (R=-0.053, p>0.05), between location 3 and the other two locations (Figure S1). That is, the isotopic signature between the pea crabs of the closest locations 1 and 2 overlap more than any of these locations with location 3 in the δ^13^C–δ^15^N biplot (see also Figure 2). A similar pattern was found in the stable isotope values of the host bivalves among locations (R=0.9, p<0.01), which were mainly due to the δ^15^N values (R=0.96, p<0.01). Although in this case the δ^13^C values also exhibited some differences between location 3 and the other two locations (R=0.65, p<0.01), but not between the closest locations 1 and 2 inhabited by the species *S. plana* and *M. galloprovincialis*, respectively (R=0.143, p=0.08). In summary, although differences in the isotopic signatures between the species *M. galloprovincialis* and *S. plana* would have been expected, the spatial variation in isotopic compositions were more evident and mainly due to the δ^15^N values. Dubois et al. (2007a, 2007b) used stable isotopes to detect small-scale changes within co-occurring trophic niches of suspension-feeding species. These studies showed significant spatial changes in δ^13^C and δ^15^N isotopic signatures in four taxonomic groups of suspension-feeders (ascidians, molluscs, polychaetes and crustaceans). However, in our study the significant effects found, through the main factors that could determine that variability (location and host species), reflected differences in the location of the aquatic systems. The effects of urban sources close to location 1, as well as the fact that the clam *S. plana* lives inside the sediment, could explain the enrichment of δ^15^N in this species. Anthropogenic nitrogen inputs into aquatic ecosystems have increased during the last decades, contributing to eutrophication in some cases (Soto et al. 2019). In any case, and in the context of this study, it is interesting to note that, regardless of the location (in the case of the mussels), the differences between the δ^15^N of the bivalves and the δ^15^N of the pea crabs were low in the two host bivalve species, suggesting that they occupy similar trophic levels.

Dubois S, Blin JL, Bouchaud B, Lefebvre S, 2007a. Isotope trophic step fractionation of suspension-feeding species: implications for food partitioning in coastal ecosystems. *J Exp Mar Biol Ecol* 351:121–128.

Dubois S, Marin Leal JC, Ropert M, Lefebvre S, 2007b. Effects of oyster farming on macrofaunal assemblages associatedwith Lanice conchilega tubeworm populations: a trophic analysis using natural stable isotopes. *Aquaculture* 271:336–349.

Soto DX, Koehler G, Wassenaar LI, Hobson KA, 2019. Spatio-temporal variation of nitrate sources to Lake Winnipeg using N and O isotope (δ^15^N, δ^18^O) analyses. *Sci Total Environ* 647: 486-493.

**Supplementary Figure S1.** Relationships between the carbon and nitrogen isotopic signal (‰) of *Afropinnotheres monodi* and the muscle of the host bivalve species *Mytilus galloprovincialis* (locations 2 and 3) and *Scrobicularia plana* (location 1) in the three locations studied. δ^13^C and δ^15^N isotopic values of the symbiont crab increased significantly, mainly δ^15^N , with those of the host bivalve.


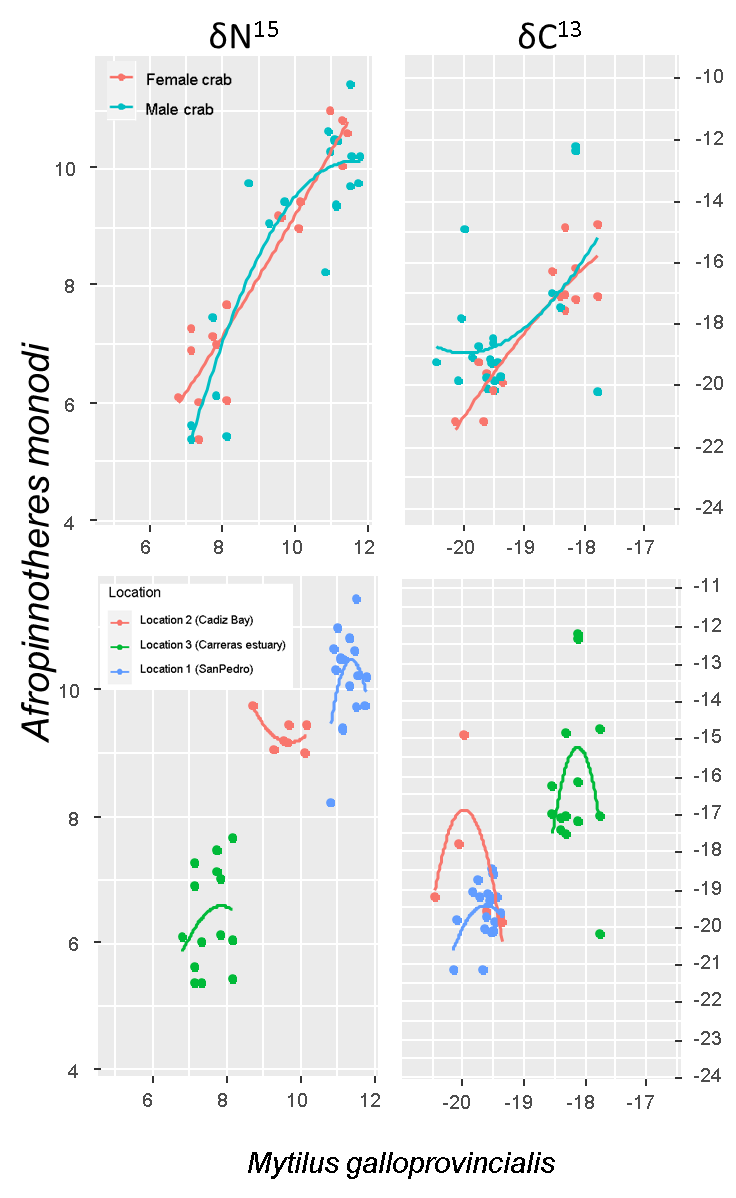

Supplement: zoab013_Supplementary_Data [file zoab013_supplementary_data.zip › zoab013-suppl_data/SUPPLEMENTARY.docx]
